# Bacteria isolated from parasitic nematodes - a potential novel vector of pathogens?

**DOI:** 10.1186/1476-069X-8-S1-S17

**Published:** 2009-12-21

**Authors:** Lizeth Lacharme-Lora, Vyv Salisbury, Tom J Humphrey, Kathryn Stafford, Sarah E Perkins

**Affiliations:** 1 School of Life Sciences, University of the West of England, Frenchay Campus, Bristol, BS16 1QY, UK; 2 Center for Infectious Disease Dynamics, Penn State University, Pennsylvania, PA 16802, USA; 3 Department of Clinical Veterinary Science, University of Bristol, Langford, BS40 5DU, Bristol, UK; 4 Cardiff School of Biosciences, Biomedical Sciences Building, Museum Avenue, Cardiff, CF10 3AX

## Abstract

Bacterial pathogens are ubiquitous in soil and water - concurrently so are free-living helminths that feed on bacteria. These helminths fall into two categories; the non-parasitic and the parasitic. The former have been the focus of previous work, finding that bacterial pathogens inside helminths are conferred survival advantages over and above bacteria alone in the environment, and that accidental ingestion of non-parasitic helminths can cause systemic infection in vertebrate hosts. Here, we determine the potential for bacteria to be associated with parasitic helminths. After culturing helminths from fecal samples obtained from livestock the external bacteria were removed. Two-hundred parasitic helminths from three different species were homogenised and the bacteria that were internal to the helminths were isolated and cultured. Eleven different bacterial isolates were found; of which eight were indentified. The bacteria identified included known human and cattle pathogens. We concluded that bacteria of livestock can be isolated in parasitic helminths and that this suggests a mechanism by which bacteria, pathogenic or otherwise, can be transmitted between individuals. The potential for helminths to play a role as pathogen vectors poses a potential livestock and human health risk. Further work is required to assess the epidemiological impact of this finding.

## Background

It is well established that bacterial pathogens can survive in soil and water for long periods and their persistence in the environment leads to increased risk of infection in human and animal hosts [1]. In 2006 fresh spinach contaminated with *Escherichia coli *was distributed throughout the USA, resulting in a multistate outbreak of food poisoning [2]. Although recent outbreaks of food-borne disease caused by *E. coli *and *Salmonella *have been well documented, and may, in part, be due to increased consumption of uncooked and organically grown vegetables, it has proved difficult to elucidate the full range of factors involved and therefore manage the risks to human health from pathogenic bacteria in the environment. One hypothesis that is under investigation is that free-living helminths, ubiquitous and numerous in the environment alongside pathogens may be a vector, or reservoir, of bacterial pathogens.

**Figure 1:**
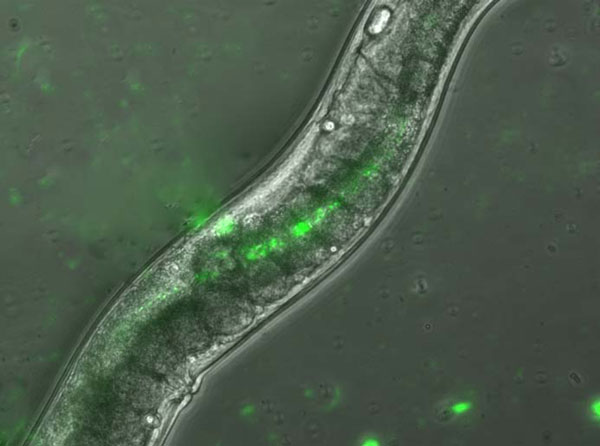
***C. elegans *showing internalized salmonella bacteria**. A fluorescent microscopy image of the free-living helminth *Ceanorhabitis elegans *that had fed, for 36 hours, on a lawn of *Salmonella *Typhimurium that are expressing green fluorescent protein (GFP), so that the bacteria emitted phosphorescent light. Bacteria were externally removed from the *C. elegans *and can be clearly seen in the intestine.

Evidence supporting the role of *Caenorhabditis elegans*, a common laboratory model system, and other free-living helminths, as potential vectors of pathogens include a number of studies that have found bacteria to survive internally to the helminth and to have increased survival [3-7], Figure 1). Additionally, ingestion of *C. elegans *has been shown to cause systemic infection in vertebrate hosts, suggesting that, given sufficient numbers, they can be vectors of food-borne pathogens [7].

Numerous and ubiquitous in the environment, alongside the non-parasitic helminths are the parasitic helminths, ranging in density from 100's to 10,000's per kilogram of dry herbage [8]. Accordingly, with these figures, helminth infection in vertebrates, especially livestock and humans in developing countries, is a pervasive health issue [9]. Parasitic helminths are more intimately associated with bacterial pathogens than the non-parasitic helminths. For example, they are often found in a host alongside a concomitant pathogen infection [10,11]. Additionally, many parasitic helminth species have a free-living stage in the environment and are in direct contact with bacterial pathogens that are excreted from infected hosts. Direct life cycle parasitic helminths are excreted with the host faeces as eggs and, after a short period of approximately a few days, develop into free-living larval helminths ready to infect a susceptible host. During this development period the helminths are associated with bacteria in the environment. Given the long evolutionary history and sympatric distribution of gastro-intestinal bacterial pathogens and parasitic helminths both inside the host and in the environment it would be surprising if helminths were not associated with pathogens [12]. To determine whether parasitic helminths act as vectors for bacterial pathogens in human and animal hosts, the first step is to investigate whether parasitic helminths carry viable pathogenic bacteria.

In this preliminary study, parasitic helminths (*Ostertagia ostertagi*, *Cooperia onchophora *and *Haemonchus contortus*) were isolated from fecal samples taken from sheep and cattle. The bacteria associated with the parasitic helminths were identified and to assess their role as potential vectors a reference was made with previous publications to determine if they have been previously denoted as pathogenic or not.

## Methods

Fecal material was collected from domestic livestock (cattle and sheep) that were known to be infected with the parasitic helminths; *Ostertagia ostertagi*, *Cooperia onchophora *and *Haemonchus contortus*. *C. onchophora *were isolated from cattle located in the South West of the UK, *O. ostertagia *were isolated from cattle in Belgium and *H. contortus *from Scottish sheep. Faeces were cultured at 25°C for seven days to allow the third stage larvae (the infectious stage) to develop from the eggs in the fecal sample. Larvae were extracted from the samples using the baermannisation method, where the fecal material is suspended over a 150 μm sieve contained in a large funnel, covered with water and left at 25°C overnight [13]. The third stage larvae (L_3_) migrate out of the fecal material and were collected in a centrifuge tube connected to the funnel. Larvae were stored at +4°C for *O. ostertagi *and *C. onchophora *species and at 12°C for *H. contortus*. Two hundred L_3 _helminths of each species were washed with M9 buffer and incubated in an antibiotic solution (1 mg/ml ampicillin/1 mg/ml gentamicin) for 1 h to kill external bacteria. After three washes with M9, worms were re-suspended in 1 ml M9 containing 0.5 g silica beads and were homogenized at 30 oscillations per second for 1 minute in a Tissue Lyser (Qiagen, Germany). Whole homogenates were plated onto blood agar plates and the colonies grown after 24 h incubation at 37°C were isolated.

Identification of bacteria recovered from inside the larvae was carried out using a Biolog Microbial Identification System (Biolog, Inc, Hayward, USA), which differentiates bacteria according to their substrate utilisation profiles [14]. Bacteria isolates were first typed by Gram staining, and then inoculated onto 0.85% saline solution at a specified optical density and subsequently inoculated onto 96-well Biolog MicroPlates (Biolog, Inc, Hayward, USA). Microplates were incubated at 37°C for 24 h and changes in absorbance at 590 nm were recorded after 4 and 24 hours of incubation. Profiles of substrate utilisation were compared with profiles in the Biolog databases of known Gram positive and negative bacteria in order to identify the isolated bacteria.

## Results

Eleven isolates were selected to carry out identification using a Biolog system; eight of which were identified (Table 1). Three isolates were not identified by the system. All three of the parasitic helminths that were cultured had bacteria associated with them. Of the eight bacteria isolated seven of them were potential human pathogens, although they ranged from rare to common pathogens. One of the bacterial isolates was a known pathogen of cattle and sheep and so potentially involved in the transmission cycle of these infections. In summary, four bacteria are known to be pathogenic to humans, of which one is also pathogenic and one commensal to livestock. Three are opportunistic human pathogens and one is both a human and cattle commensal (Table 1).

**Table 1:** Bacteria isolated from helminths cultured from feaces samples of livestock. A list of bacteria isolated from three different species of parasitic helminths. An indication of whether the bacteria are pathogenic or commensal and the type of infection they produce are given.

Bacteria	Helminth	Host	Comments
*Salmonella *gp 3B (diarizonae)	*Cooperia onchophora*	Cattle	Human and animal pathogen - found in sheep, cattle and horses.
*Corynebacterium auris*	*Cooperia onchophora*	Cattle	Unknown pathogenicity in cattle. Occasionally isolated from ear infections in humans.
*Bacillus pumilus C*	*Cooperia onchophora*	Cattle	Cattle commensal. Rare human pathogen.
CDC group II-E subgroup A	*Ostertagia ostertagi*	Cattle	Unknown pathogenicity in sheep. Pathogenic for humans - found in the hospital environment.
*Tsukamurella inchonensis*	*Ostertagia ostertagi*	Cattle	Unknown pathogenicity in sheep. Opportunistic human pathogen common in soil and water.
*Rahnella aquatilis*	*Ostertagia ostertagi*	Cattle	Unknown pathogenicity in sheep. Opportunistic human pathogen, found in water.
*Sphingobacterium multivorum*	*Haemonchus contortus*	Sheep	Unknown pathogenicity in sheep. Opportunistic human pathogen, isolated from soil, plants, and water
*Streptococcus macacae*	*Haemonchus contortus*	Sheep	Sheep commensal. Human commensal.

## Discussion and conclusion

This study was a qualitative survey to show proof of principle that parasitic helminths may act as vectors for viable bacteria and, in the case of pathogens, present a reservoir or vector for the transmission of pathogens between hosts. The bacteria isolated from the helminths included one pathogen of both cattle and sheep, although the pathogenicity to livestock of most isolates was unknown [15]. Regardless of whether the bacterial isolates were pathogens or commensals it remains that bacteria can survive within helminths. As such, helminths may provide an additional method of pathogen transmission between hosts and may confer protection from the environment to the bacteria, in a manner similar to observations upon free-living non-parasitic helminths [4,7].

Previous evidence of parasitic helminths acting as vectors does exist. Parasitic helminths of plants are well known vectors of a range of plant pathogens, but less studied are examples of parasitic helminths of vertebrates as vectors of pathogens (but see [16] for a review). The discovery of plant helminth vectors has prompted some experimental work with helminths of vertebrate hosts, and facultative vectoring of pathogens through experimental infection of naive hosts with pathogen-infected helminths was documented [17]. Additionally, *Mycobacterium avium *has been isolated from helminths cultured from fecal samples of hosts that were simultaneously co-infected with both helminths and *Mycobacterium avium *[18]. Obligate vectoring, where transmission is due entirely to the helminth, has also been implicated for a few pathogens, interestingly in aquatic environments [19,20]. Despite evidence that parasitic helminths can harbour pathogens and that these can lead to host infection there is a paucity of studies that have investigated the range of bacterial pathogens associated with parasitic helminths. Considering how common helminth infection is and the consequences for host health of helminth vectoring there is a clear need for further studies of this type [16].

We have shown a range of animal (and human) pathogens are found internally to different parasitic helminth species that were cultured from host feaces, indicating that they have the potential to vector bacteria to their hosts, or to perpetuate bacteria in the environment. However, the presence of bacteria in the parasitic helminths is only an indication of their vectorial capacity. To fully understand their contribution to pathogen transmission Koch's postulates must be fulfilled. As such, the next step would involve determining whether infection occurs after ingestion of bacteria-infected helminths by naïve hosts. This study raises a series of questions - were the bacteria acquired from the fecal material in which the helminths were cultured? Or were the pathogens present in the helminth eggs? Either way helminths may act as vectors by perpetuating infection within an individual or in transmitting pathogens between susceptible hosts. Ascertaining the diversity, quantity and survival of bacterial pathogens that are within parasitic helminths as well as their capacity to cause infection is a clear research priority.

## Note

The peer review of this article can be found in Additional file 1.

## Competing interests

The authors declare that they have no competing interests.

## Authors' contributions

VS, SP, TH and LLL contributed equally to the idea and paper writing. KS collected and cultured the parasitic helminths.

## Supplementary Material

Additional file 1 Peer review. Click here for file
