# Supplementary material for: Bacteria isolated from parasitic nematodes - a potential novel vector of pathogens?
Source: Environ Health. 2009 Dec 21;8(Suppl 1):S17. doi: 10.1186/1476-069X-8-S1-S17 (PMC2796495; doi:10.1186/1476-069X-8-S1-S17)
Supplement: Additional file 1 — Peer review. [file 1476-069X-8-S1-S17-S1.doc]

| Article ref no. | A1471-2105X SZ-S17 |
| --- | --- |
| Title | **Bacteria isolated from parasitic nematodes – a potential novel vector of pathogens?** |
| Author(s) | Lacharme-Lora et al. |

**Ref#1**

**General comments:**

Abstract line 12 – pathogens

Table 2 full stop after ‘Unknown pathogenicity in cattle’.

             Full stop after ‘Cattle commensal’ and other sentences.

            Cattle not sheep are the normal host for *Ostertagia ostertagi*

# Ref#2

Well written paper that conveys exciting preliminary data with far reaching implications for animal husbandry and public health.

Recommend acceptance after minor corrections:

Abstract Line12 pathogens

Page 6 Line 12 bacterial not bacteria

# Response to Referees’ comments

All changes have been made as recommended.
